# Au Ordered Array Substrate for Rapid Detection and Precise Identification of Etomidate in E-Liquid Through Surface-Enhanced Raman Spectroscopy

**DOI:** 10.3390/nano14231958

**Published:** 2024-12-06

**Authors:** Yan Mo, Xiaoping Zhang, Ke Zou, Wen Xing, Xiayang Hou, Yu Zeng, Yugang Cai, Ruixiang Xu, Hongwen Zhang, Weiping Cai

**Affiliations:** 1 Intelligent Policing Key Laboratory of Sichuan Province, Sichuan Police College, Luzhou 646000, China; moyan6868@scpolicec.edu.cn (Y.M.); houxy0816@yeah.net (X.H.); prxd741852@163.com (R.X.); 2 Anhui Xianghe Environmental Testing Co., Ltd., Huaibei 235100, China; m15105613166@163.com (X.Z.); zouke3003@outlook.com (K.Z.); xingwen_4875@163.com (W.X.); 3 Department of Intelligent Manufacturing and Automotive Engineering, Luzhou Vocational & Technical College, Luzhou 646000, China; muwanziyu7000@163.com; 4 Luzhou Public Security Bureau, Luzhou 646000, China; caiyugang01@126.com; 5 Key Laboratory of Materials Physics, Anhui Key Laboratory of Nanomaterials and Nanotechnology, Institute of Solid State Physics, HFIPS, Chinese Academy of Sciences, Hefei 230031, China; wpcai@issp.ac.cn; 6 Key Laboratory of Toxic and Harmful Gas Monitoring and Early Warning, Ministry of Emergency Management, Baoding 065201, China

**Keywords:** surface-enhanced Raman spectroscopy, SERS, Au ordered array, etomidate, e-liquid, rapid drug detection

## Abstract

Etomidate (ET), a medical anesthetic, is increasingly being incorporated into e-liquids for consumption and abuse as a new psychoactive substance (NPS), leading to significant social issues. In this work, large-area Au micro- and nano-structured ordered arrays were engineered as surface-enhanced Raman spectroscopy (SERS) substrates for fast detection and precise identification of ET and its metabolites. This ordered array, characterized by abundant electromagnetic enhancement hotspots and structural uniformity, imparts unique properties to the SERS substrate, including ultra-sensitivity, spectral signal reproducibility, and precise quantitative capabilities. Furthermore, it effectively mitigates interference from the complex matrix of e-liquids, facilitating the rapid detection of trace amounts of ET molecules. This SERS rapid detection technology can act as a preliminary screening method for gold-standard spectroscopic analysis, facilitating the on-site rapid screening of suspicious samples and thereby enabling efficient detection and precise verification.

## 1. Introduction

Etomidate (ET) is a commonly used intravenous anesthetic that belongs to the imidazole class of drugs and is classified as a new psychoactive substance (NPS) [1]. It produces sedation and anesthesia by inhibiting γ-aminobutyric acid (GABA) metabolism in the brain and enhancing the inhibitory effect of GABA on the nervous system [2]. According to investigations, ET has become a drug substitute for some people who formerly abused drugs, and a number of abuse cases have been found in which ET was added to e-liquid oil [3,4]. In some cases, ET abuse has even caused deaths [5,6]. A psychiatric research inquiry into 560 cases of individuals with ET abuse found that the primary carrier form of abuse was e-liquid, in which 103 patients had impulsive aggression and other dependence syndromes and 150 cases were accompanied by broken bones. The abuse of ET e-liquid is very serious and shows a trend in a growing group of adolescents (drug abuse by minors was 16% in the study mentioned above). In 2023, China uncovered 8667 cases involving ET and arrested 68,000 ET abusers. Due to its strong addictive features, vicious cases caused by the abuse of ET, such as self-injury, suicide, traffic accidents, rape, and so on, have occurred from time to time. Its harmfulness and abuse have aroused wide attention and serious social problems [7].

The development of the ET detection method provides strong technical support for drug control work. To our knowledge, research on ET has been based on the analysis of blood [8], urine [9], hair [10,11], and other biological materials [12]. Wastewater analysis can also provide information about and comparisons of differences in ET consumption trends across time and space; Shuai Yuan et al. [13] used ultra-performance liquid chromatography–tandem mass spectrometry to monitor the use of ET in wastewater samples from several wastewater treatment plants, and satisfactory limits of detection were obtained. It is not difficult to see that the detection methods in these investigations are mainly applied to laboratory testing, and the methods used mainly focus on liquid chromatography–mass spectrometry (LC-MS) techniques, which show favorable testing results for ET and even its metabolites.

But for anti-narcotic drug work, relying on the rapid detection of ET at the scene of the case is also very important. Therefore, rapid and sensitive detection of ET is of great significance. Jiahao Li et al. [14] reported a novel fluorescent sensing probe which presented a high precision linear relationship for ET in electronic cigarette liquids, and the whole detection process was rapid, simple, and easy to operate. Lijuan Huang et al. [15] developed a simple optical sensor array for the classification of nine narcotic drugs in aqueous solution and human urine, and the proposed method had potential for on-site drug detection and drug abuse screening. Yutian Zhao et al. [16] presented a fluorescent supramolecular sensor, namely, an albumin-based indicator displacement assay, for the first time, which successfully achieved the ratiometric determination of ET with a discernible color response.

In addition, Raman spectroscopy and surface-enhanced Raman spectroscopy (SERS) are also widely used in the rapid detection of illicit drugs due to their simple operation, fast response, and fingerprint identification [17,18,19,20]. The method was successfully applied to the rapid screening of cocaine, amphetamine, cannabinol, fentanyl, cannabinoid, morphine, tramadol, phencyclidine, methadone, methamphetamine, etc. [21,22,23,24,25,26]. Ikechukwu C. Nwaneshiudu et al. [27] used polydimethylsiloxane-based solid-phase micro-extraction, along with Raman spectroscopy, to separate and enhance the detection of ET from aqueous and serum phases. However, in general, rapid test methods for ET are still scarce, and there are few reports on the rapid detection of ET by the SERS method at present.

Based on this, this work proposed a method for rapid detection and precise identification of ET in e-liquid by using a gold (Au) micro/nano-structured array as a SERS substrate. The numerous gaps between the nanoparticles generate a substantial number of electromagnetic enhancement hotspots for the SERS detection of ET molecules. Moreover, their long-range ordered array structure guarantees the reproducibility of Raman signals, thereby enabling reliable quantitative detection. This SERS technology can be employed for the rapid on-site detection of ET drugs in e-liquid, offering a convenient and efficient approach for drug enforcement.

## 2. Experimental Section

### 2.1. Materials and Reagents

Silicon (Si) wafers were obtained from Zhejiang Kaihua Jingxin Electronics Co., Ltd. (Quzhou, China). A water suspension of mono-dispersed polystyrene (PS) microspheres with a diameter of 120 nm and 2.5 wt.% was sourced from Shanghai Aladdin Biochemical Technology Co., Ltd. (Shanghai, China), while 4-mercaptobenzoic acid (4-MBA) of 95% purity, methanol of chromatographic purity, and ethanol of analytical purity were purchased from Sinopharm Chemical Reagent Co., Ltd. (Shanghai, China). Standard solutions of ET and etomidate acid (ETA) in methanol were procured from Beijing Spectrum Analytical Standard Technology. E-cigarette oil was acquired from an authorized store. Deionized water with a resistivity of 18.2 MΩ cm was obtained from an ultrafilter system (Millipore Milli-Q system, Marlborough, MA, USA). All reagents were employed without additional purification.

### 2.2. Construction of High-Density Gold Micro/Nano-Arrayed SERS Substrates

**Figure 1:**
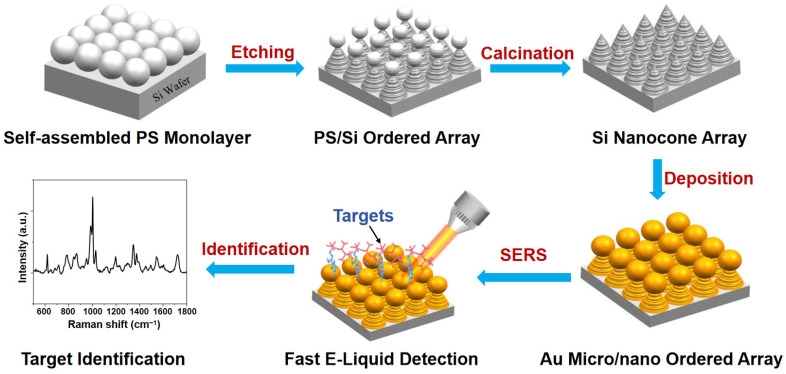
Schematic diagram of template-based fabrication of Au micro- and nano-structured ordered arrays as sensitive SERS substrate for fast E-liquid detection and drug identification.

Au micro/nano-arrayed SERS substrates were readily prepared through template-assisted etching and deposition method (Figure 1). Using the gas–liquid interface self-assembly method, a monolayer of PS colloidal template was prepared on a 3 cm × 3 cm Si substrate. Initially, deionized water was carefully applied to the clean Si substrate surface, followed by the controlled dripping of PS sphere liquid from the edge. After achieving dynamic equilibrium and obtaining a monolayer floating on the liquid surface, filter paper was used to absorb excess water from the edge of the substrate, and then allowed to air dry naturally. Subsequently, the monolayer-covered Si substrate was introduced into a reactive ion etching machine with a vacuum cavity set at 2.0 Pa. Etching gas composed of 30 sccm/min SF_6_ and 10 sccm/min O_2_ was passed through during the process, with the substrate etched at 200 W for 30 s to obtain the Si nanocone array. The residual PS spheres were removed by subsequent calcination at 400 °C in a muffle furnace under an air atmosphere for 2 h. A magnetron sputtering instrument (EMITECH K550X, Quorum Technologies, Kent, UK) was employed to apply a large current of 30 mA for 4 min to the surface of the Si nanocones for sputter deposition, thereby generating a surface-rough Au micro/nano-arrayed substrate. For comparative purposes, Au nanoparticle films were prepared on a smooth Si substrate under identical sputtering conditions.

### 2.3. Theoretical Simulation

The electric field distribution on the surface of the nanoparticles was calculated by FDTD theoretical simulation under 785 nm wavelength laser excitation. The Raman spectra of the ET molecule were simulated with density functional theory (DFT) by the Gaussian 09 package.

### 2.4. Characterization

The Au substrates were cut into 2 mm × 2 mm pieces for subsequent characterization and SERS evaluation. The surface and profile morphology were examined utilizing a Sigma 300 Field Emission Scanning Electron Microscope (Zeiss, Jena, Germany). Elemental composition and distribution were analyzed using an XFlash 6/30 Energy Spectrometer (Bruker, Karlsruhe, Germany). The SERS experiments were performed using a portable Raman spectrometer (*i*-Raman, BWS415-785S, BWTEK, Newark, USA). The laser power was consistently set at 32 mW, which corresponds to 10% of the total laser energy. The integration time was established at 5 s, with a repetition count of 1. All the acquired spectral data were subjected to baseline correction and smoothing using the BWSpec software (BWT-810000044) provided with the instrument.

## 3. Results and Discussion

### 3.1. Morphology and Structure

**Figure 2:**
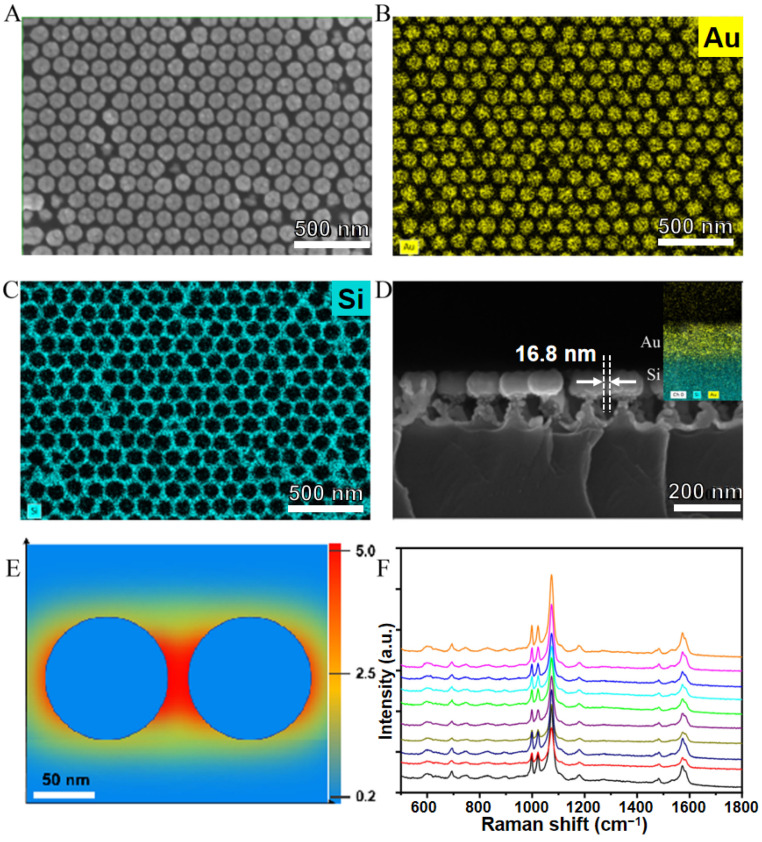
(**A**) SEM image of the SERS substrate composed of Au ordered array. (**B**,**C**) The EDS analysis of the Au and Si elemental distribution in the SERS substrate. (**D**) The SEM cross-sectional observation of the substrate, along with the elemental distribution. (**E**) The theoretical simulation of the spatial electric field distribution of the structural unit of the array (sphere size 100 nm, gap distance 15 nm). (**F**) SERS spectra of 10^−7^ M 4-MBA probe molecule and its spectral reproducibility. Each color curve corresponds to a spectrum acquisition point.

When using Au nanoparticles as SERS materials, we generally consider their optimal size to be 50–100 nm [28]. Sizes that are too large or too small can reduce the inherent Raman enhancement effect of the nanoparticles. In this work, the surface roughness and size of the basic unit of the SERS substrate could be precisely controlled by adjusting the sputtering current and sputtering time. Typically, a larger sputtering current results in a rougher surface, while a longer sputtering time increases particle size and reduces the gaps between the spheres. Figure 2 illustrates the microscopic morphology and compositional characteristics of the SERS substrate. The basic structural unit of the substrate consists of micro- and nano-structured spherical structures, approximately 100 nm in size, which form a long-range ordered array through a hexagonally close-packed arrangement (Figure 2A). EDS surface elemental analysis (Figure 2B–D) reveals that these structural units are primarily composed of Au element. Cross-sectional analysis indicates that the nearly spherical Au nanoparticles are positioned at the tops of silicon cones, with an interparticle gap distance of approximately 16.8 nm. Compared to the smooth Au nanocone array [29], this ordered array, which uses rough nanospheres as structural units, is obtained through a greater sputtering current and extended sputtering time. Such a rough surface and long-range ordered array contains numerous electromagnetic enhancement “hotspots” that contribute to elevated Raman activity. Employing 4-MBA as the probe molecule at a concentration of 10^−7^ M, we obtained a complete molecular vibration spectrum characterized by distinct and sharp peaks (Figure 2F). The definition of the SERS enhancement factor (EF) is shown in Equation (1) [30]:

(1) $$EF=\frac{I_{SERS}/N_{Surf}}{I_{RS}/N_{Vol}}$$

where I_SERS_ and I_RS_ are the scattering intensities of SERS and normal Raman scattering, respectively; N_Surf_ is the number of molecules adsorbed onto the SERS substrate in the area being probed; and N_vol_ is the number of molecules in the excitation volume of the laser used in normal Raman-scattering measurements. The EF of the SERS substrate was calculated as 3.26 × 10^7^, indicating that its ultra-sensitive characteristics hold promise for the trace detection of target molecules. Additionally, the structural uniformity facilitated reproducible SERS spectra during random detection, with a relative standard deviation (RSD) of less than 5.0%. Consequently, when employed for the identification and detection of ET, it is anticipated to generate ultrasensitive and reliable characteristic SERS signals.

### 3.2. SERS Detection and Identification of ET 

#### 3.2.1. Sensitive SERS Detection and Assignment of Vibrational Peaks

**Figure 3:**
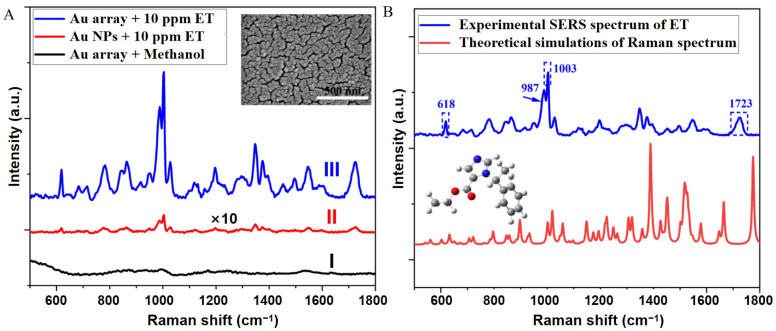
(**A**) SERS spectrum of ET at a concentration of 10 ppm acquired from the Au nano-structured ordered array substrate. The comparison sample is Au nanoparticle (NP) film obtained by sputter deposition with identical parameters. (**B**) Theoretically simulated Raman spectrum of ET molecules, used to assign the characteristic vibrational peaks in the SERS spectrum.

In contrast to the colloidal solutions of noble metals produced via chemical synthesis methods, the Au nano-structured ordered arrays obtained through physical sputtering demonstrate exceptional surface cleanliness. As illustrated in Curve I of Figure 3A, upon applying chromatographically pure methanol solution to the surface and allowing it to dry, no characteristic peaks were observed during SERS analysis. This high level of surface cleanliness not only effectively eliminates background peak interference, but also mitigates competitive adsorption of molecules on the surface, thereby enabling the full utilization of electromagnetic enhancement hotspots for the detection of target molecules [31,32]. Consequently, when detecting 10 ppm ET molecules using the Au ordered array substrate, a pronounced SERS spectrum was obtained, characterized by rich and sharp vibrational peaks (Curve III). In comparison to the Au nanoparticle (NP) film prepared by sputter deposition with identical parameters (Curve II), the intensity of the characteristic peak at a wavenumber of 1003 cm^−1^ increased by two orders of magnitude.

To assign the vibrational peaks in the SERS spectrum, we conducted theoretical simulations of the Raman spectrum of ET molecules using DFT (Figure 3B). It is concluded that the two strongest characteristic peaks located at 1003 cm^−1^ and 987 cm^−1^ are primarily attributed to the breathing vibration of the benzene ring and the stretching vibration of C–CH_3_ in heterocycle, respectively. Moreover, the peak at 1723 cm^−1^ belonged to the C=O stretching vibration of α, β unsaturated esters, and the rest of the peaks at 618, 785, 851, 1200, 1351, and 1375 cm^−1^ are closer to the Raman peaks reported in the literature (Table 1) [27]. This demonstrates that the SERS spectrum of the ET molecule possesses a high degree of similarity in spectral shape to the Raman spectrum; however, the number of characteristic peaks in the SERS spectrum is significantly lower than that in the Raman spectrum. This discrepancy arises from the stable adsorption of the ET molecule onto the gold substrate through strong interactions, whereby only the vibrational modes near the gold surface can be effectively enhanced.

#### 3.2.2. Reproducibility and Quantitative Detection

**Figure 4:**
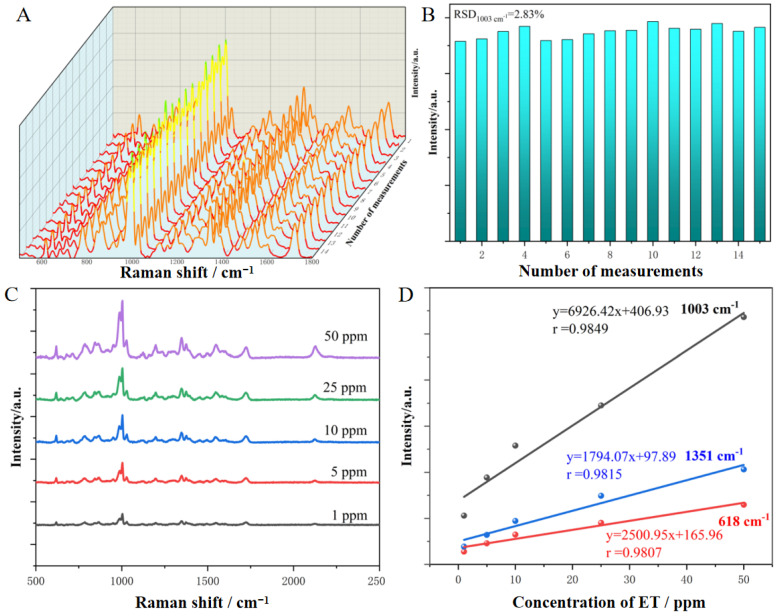
(**A**) Reproducibility of SERS spectra for 10 ppm ET molecules acquired from one substrate. (**B**) The intensity and relative standard deviation (RSD) of the vibrational peak at 1003 cm^−1^ within and between SERS substrates. (**C**) SERS spectra of ET molecules with concentrations ranging from 1 to 50 ppm. (**D**) The linear relationship between the peak intensities at 618, 1003, and 1351 cm^−1^ and the concentration.

The ultra-sensitive characteristic of the SERS technique presents challenges for the reproducibility and reliability of its spectral signals; however, these factors are essential for achieving qualitative identification and quantitative detection. Figure 4A presents the Raman spectra obtained from 15 randomly selected target regions on the surface of the SERS substrate. The spectral profile and peak intensities of the different spectra exhibit a high degree of consistency. An intensity variation analysis of the characteristic peak at a wavenumber of 1003 cm^−1^ yields a relative standard deviation (RSD) value as low as 2.83% (Figure 4B), significantly lower than previously reported values in the literature [33,34,35]. Furthermore, for different SERS substrates, the RSD value is evaluated as low as 4.26%. Therefore, high signal reproducibility can be achieved both within and between substrates. This exceptional feature arises from the nanoscale structural units and long-range ordered array structure of the SERS substrate, in which the uniform distribution of hotspots ensures that a similar number of hotspots are covered when changing sampling positions. Given the high reliability of SERS spectral signals, there is potential for further quantitative detection. Figure 4C displays the SERS spectra obtained from a range of concentrations (1–50 ppm) of ET molecules. Even at a low concentration of 1 ppm, each characteristic peak of the ET molecule can still be clearly identified, confirming the high sensitivity of the SERS substrate, which meets the demands for trace drug detection. As the concentration increases, the intensities of the characteristic peaks of the ET molecules are correspondingly enhanced. The peak shapes at 618, 1003, and 1351 cm^−1^ are more prominent, so the peaks at these displacements were chosen for further quantitative detection. All corresponding calibration curves between peak intensities and ET concentrations reveal perfect linear relationships with a correlation coefficient R^2^ > 0.96 (Figure 4D). According to the limit of detection (LOD) equation (LOD = *k* × σ/S), where σ is the standard deviation of the blank for multiple measurements, S is the method sensitivity, i.e., the slope of the calibration curve (1003 cm^−1^), and *k* is a constant selected based on the desired confidence level, (the IUPAC suggests *k* = 3), the LOD was calculated to be about 17 ppb. It is essential to recognize that the linear concentration relationships obtained for different characteristic peaks exhibit variability. The main reason is that SERS is a near-field enhancement effect, where the enhancement of vibrational peaks near the substrate surface is pronounced and gradually diminishes with increasing distance from the substrate. When the concentration of molecules changes, their adsorption states are influenced by the occupation effect, resulting in variations in the intensity of different characteristic peaks. Consequently, the Au ordered array substrate enables reliable qualitative identification and precise quantitative detection of trace ET molecules.

### 3.3. Rapid SERS Detection of ET Drugs in E-Liquid

**Figure 5:**
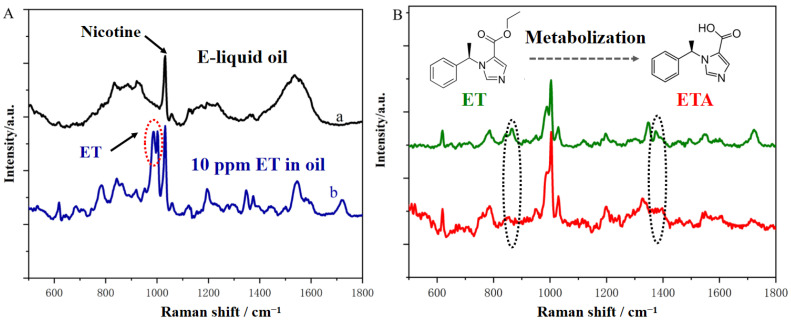
(**A**) SERS spectra of e-liquid oil and ET-containing sample with a concentration of 10 ppm. The dotted circle marks the most significant characteristic vibrational peaks that are exclusive to the ET molecule. (**B**) Spectral comparison of the SERS spectra of ET and its metabolite etomidate acid (ETA). The dotted cycles highlight the differences in the characteristic vibrational peaks of the two molecules.

In typical scenarios, actual samples possess complex matrices that can interfere with the SERS detection of target molecules, potentially resulting in a complete inability to identify the target substances. ET is often added to e-cigarette oils for illicit use. In the present work, commercially available e-cigarette oil was diluted 1000 times (*v*/*v*) with anhydrous ethanol, and then a certain concentration (10 ppm) of ET was added to investigate the actual detection ability of the as-prepared Au ordered array substrates. As shown in Figure 5A, the interference within the e-liquid matrix primarily stems from the presence of nicotine, whose strongest vibrational peak is predominantly situated to the right of 1000 cm^−1^, overlapping with the characteristic peak of ET molecules at 1351 cm^−1^. Aside from this peak, all other peaks can be employed for the precise identification of ET molecules.

To verify the accuracy of the quantitative results, we prepared ET target solutions at concentrations of 10 and 25 ppm, using diluted e-liquid as the matrix. We acquired the SERS spectra through the Au substrate and derived the quantitative detection results based on the previously established intensity–concentration linear relationship, utilizing the intensity of the characteristic peak at 1003 cm^−1^, and calculated the recovery rate. As described in Table 2, the recovery rate ranges from 94.7 to 104.1%, indicating the accuracy of the method for detecting ET in practical samples. The RSD of the recovery rate was less than 5%, proving that this SERS Au/Si array has great potential to achieve rapid detection of ET.

Upon inhalation through e-liquid, ET is metabolized into etomidate acid (ETA) within the human body. Similarly, we assessed the SERS detection capabilities of the as-prepared Au ordered array substrate for ETA. As illustrated in Figure 5B, the spectral profile and primary characteristic peaks of ETA closely resemble those of ET. This similarity arises because the transformation from ET to ETA does not significantly alter the molecules’ characteristic vibrations at the microscopic level; however, they can still be differentiated by the distinct characteristic peaks at approximately 850 and 1360 cm^−1^. Consequently, the ordered Au array substrate exhibits sensitive detection capabilities for both ET and its metabolites, making it suitable for detecting ET in suspicious e-liquids, as well as for testing whether individuals have consumed ET, using body fluids as samples.

## 4. Conclusions

In summary, we utilized a monolayer polystyrene (PS) microsphere array as a template, optimizing both ion etching and physical sputtering processes to create Au micro/nano-structured ordered arrays characterized by rich electromagnetic enhancement hotspots and a uniform structure. When employed as a SERS substrate for the trace detection of the ET drug, this array demonstrates exceptional sensitivity, reproducibility, and quantitative capabilities. During the analysis of actual e-liquid samples, we successfully identified and detected illicit drugs and their metabolites without the need for complex pretreatment. This SERS rapid detection technology, when efficiently integrated with gold-standard spectroscopic analysis techniques, will facilitate on-site rapid screening and precise verification of suspicious samples drawn from many specimens.

## Figures and Tables

**Table 1:** Vibrational peak assignment of ET molecule.

Peak Position/Cal. (cm^−1^)	Peak Position/Exp. (cm^−1^)	Assignment
632	618	The twist vibration of the benzene ring
796	785	The breathing vibration of the benzene ring
846	851	The umbrella vibration of C=O
1001	987	The stretching vibration of C-CH_3_ in heterocycle
1019	1003	The breathing vibration of the benzene ring
1217	1200	The C-H bending vibration of the benzene ring
1388	1351	The breathing vibration of heterocycles
1453	1375	The C-H umbrella vibration of CH_3_-CH_2_
1521	1632	The C-H umbrella vibration of the benzene ring
1774	1723	The C=O stretching vibration

**Table 2:** Detailed detection results of ET-contaminated e-liquid oil samples by SERS methods.

Spiked Concentration/ppm	Calculated Concentration/ppm		Recovery Rate/%
10	1	10.106	101.1
	2	10.412	104.1
	3	9.466	94.7
	Average	9.995	100.0
25	1	24.195	96.8
	2	25.367	101.5
	3	24.651	98.6
	Average	24.738	99.0

## Data Availability

No new data were created or analyzed in this study. Data sharing is not applicable to this article.
